# Jagged2a-Notch Signaling Mediates Cell Fate Choice in the Zebrafish Pronephric Duct

**DOI:** 10.1371/journal.pgen.0030018

**Published:** 2007-01-26

**Authors:** Ming Ma, Yun-Jin Jiang

**Affiliations:** Laboratory of Developmental Signalling and Patterning, Institute of Molecular and Cell Biology, Singapore, Singapore; University of Pennsylvania School of Medicine, United States of America

## Abstract

Pronephros, a developmental model for adult mammalian kidneys (metanephros) and a functional kidney in early teleosts, consists of glomerulus, tubule, and duct. These structural and functional elements are responsible for different kidney functions, e.g., blood filtration, waste extraction, salt recovery, and water balance. During pronephros organogenesis, cell differentiation is a key step in generating different cell types in specific locations to accomplish designated functions. However, it is poorly understood what molecules regulate the differentiation of different cell types in different parts of the kidney. Two types of epithelial cells, multi-cilia cells and principal cells, are found in the epithelia of the zebrafish distal pronephric duct. While the former is characterized by at least 15 apically localized cilia and expresses *centrin2* and *rfx2,* the latter is characterized by a single primary cilium and sodium pumps. Multi-cilia cells and principal cells differentiate from 17.5 hours post-fertilization onwards in a mosaic pattern. Jagged2a-Notch1a/Notch3-Her9 is responsible for specification and patterning of these two cell types through a lateral inhibition mechanism. Furthermore, multi-cilia cell hyperplasia was observed in *mind bomb* mutants and Mind bomb was shown to interact with Jagged2a and facilitate its internalization. Taken together, our findings add a new paradigm of Notch signaling in kidney development, namely, that Jagged2a-Notch signaling modulates cell fate choice in a nephric segment, the distal pronephric duct.

## Author Summary

The kidney is a complex organ that regulates blood homeostasis through the maintenance of fluid and ion balance and disposal of metabolic waste. We used zebrafish pronephros, a primordial vertebrate kidney, to address how a kidney tissue acquires its cell types and pattern. Two types of epithelial cells were found in the pronephric duct: multi-cilia cells and principal cells, which could be distinguished based on morphology and expression of different marker genes. In the pronephric duct, the multi-cilia cells and principal cells form a “salt and pepper,” or mosaic, pattern. Using existing zebrafish mutants and a knockdown technique, we demonstrated that the mosaic pattern and differentiation of these two cell types are controlled through a Notch-dependent lateral inhibition mechanism. Notch signaling has been shown to be essential for other aspects of kidney development, such as formation of the glomerulus and the tubule. Here, to our knowledge for the first time, we show that the same signaling pathway is required for the differentiation of two different epithelial cells in a kidney segment known as the distal pronephric duct. The same mechanism is very likely to be employed by other similar developmental processes in the same context to generate distinct cell types in a tissue.

## Introduction

In vertebrates, development of the excretory system is characterized by the successive formation of three distinct kidneys with increased complexity: pronephros, mesonephros, and metanephros. The pronephros is found in all vertebrates, but in mammals it is a nonfunctional transitory structure that is replaced by the mesonephros and then the metanephros. In the early life of fish and amphibians, however, the pronephros is a functional filtration organ that develops very similarly to metanephros, and has been used as a model for kidney development. Nephrons, the fundamental functional units of the kidney, possess several segments, which regulate fluid balance, osmolarity, and the disposal of metabolic waste products [1]. While pronephroi in amphibians and fish contain two functional nephrons, the metanephroi of mammals have millions of nephrons [1–3]. The zebrafish pronephros consists of paired glomeruli coalescing at the midline ventral to the dorsal aorta, and two pronephric tubules that project bilaterally from the glomeruli to the pronephric (Wolffian) ducts that run caudally and fuse just before their contact with the exterior at the cloaca [2]. The glomerulus is the site of blood filtration. Epithelia of the tubules are the primary site of selective reabsorption and secretion, while the duct carries the modified urine to the outside world [1]. Though quite uniform in appearance, the tubule and duct epithelia are further subdivided into distinct segments, recognized by the expression of specific membrane transporters [4]. This is a general feature of vertebrate kidneys, where osmoregulatory function depends on an organized disposition of different transporters operating sequentially along the nephron [5–7].

Morphogenesis and cell fate determination of different nephric segments have attracted much attention recently. Multiple transcription factors and signaling pathways have been shown to be involved in these processes in different model organisms. Wnt4 is essential for tubulogenesis in mouse metanephroi and *Xenopus* pronephroi [8,9]. Brn1 is required for the development of Henle's loop, the distal convoluted tubule, and the macula densa in mice at the primitive loop stage [10], and so is *pax2a* for the differentiation of proximal tubule and duct epithelial cells and cloaca morphogenesis in zebrafish [11]. Some segments of the nephron comprise only one cell type, while others include two or more cell types. The mammalian collecting duct contains two major cell types: principal cells (for salt and water absorption) and intercalated cells (for acid/base transport) [12]. It was reported that *Foxi1* plays a crucial role in the specification of intercalated cells [13].

Notch signaling is an evolutionarily conserved pathway that multicellular animals use in regulating pattern formation and cell fate determination through local cell interactions [14,15]. One of the well-known mechanisms of Notch signaling is lateral inhibition during neurogenesis: initially equivalent cells differentiate into a “salt and pepper” pattern of cells with different fates via a regulatory loop [14]. Notch is a transmembrane receptor that interacts with Delta and Serrate/Jagged ligands. Ligand-activated intramembrane proteolysis, which is partly through the γ-secretase activity of Presenilin, is required to release the Notch intracellular domain (Notch^icd^), which is then translocated to the nucleus, where Notch^icd^ and CSL (CBF1/RBPjκ, Su(H), and Lag-1) proteins bind and activate downstream target genes, such as *Hairy*/*Enhancer of split related* (*Hes*/*her*) homologs [16]. Ubiquitylation is a multistep process that results in the conjugation of Ubiquitin to a substrate protein. Recent studies have identified the roles of Neuralized and Mind bomb (Mib) in ligand ubiquitylation and endocytosis, which is essential for activating Notch [17–20]. Similarly, Jagged2 is ubiquitylated by a Mib paralog, Skeletrophin [21].

Notch signaling is required for the development of different kidney segments. By manipulating Notch activity, Notch signaling in the tubule was shown to inhibit duct fate in the dorsoanterior *Xenopus* pronephric anlage and to control subsequent tubule patterning [22]. Homozygous *Notch2^del1^,* a hypomorphic allele, and transheterozygous *Notch2^del1^*/*Jag1^dDSL^* mice exhibit a similar glomerular defect: lack of capillary tufts and mesangial cells [23]. Presenilin is indispensable for the formation of mouse proximal tubules and glomerular podocytes [24,25]. Zebrafish *jagged1b*/*jagged2a* double morphants have small glomeruli or segments of glomerulus replaced by dilated blood vessels [26].

Our analysis of zebrafish pronephric ducts revealed that the distal ducts are composed of two types of epithelial cells: multi-cilia cells and principal cells. We showed that multi-cilia cells interpolate principal cells and that their differentiation is mediated by Jagged2a-Notch1a/Notch3-Her9 signaling. We also demonstrated that this differentiation process requires Mib, an E3 ligase that facilitates Jagged2a endocytosis, and, hence, activates Notch signaling. This is the first time, to our knowledge, that Jagged2-Notch signaling has been shown to mediate cell fate determination within a kidney segment, but not between segments, via a lateral inhibition mechanism.

## Results

### Multi-Cilia Cells Interpolate Principal Cells in the Zebrafish Pronephric Duct

**Figure 1:**
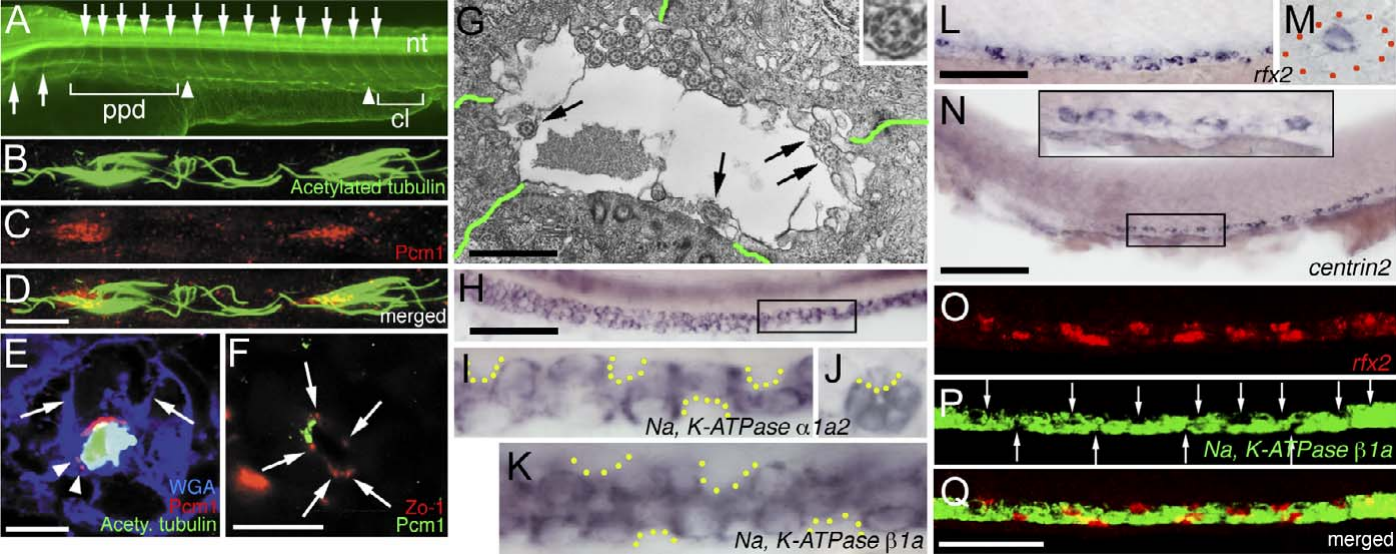
Multi-Cilia Cells and Principal Cells Interpolate in the Pronephric Duct Embryo in (A) is 48 hpf, embryos in (O–Q) are 27 hpf, and all others are 36 hpf. (A) Acetylated tubulin staining revealed that cilia tufts are located in the lumen of the distal (demarcated by arrowheads) but not proximal pronephric duct (ppd) or cloaca (cl). Arrows point to the ventral axons of caudal primary motor neurons (CaP), which project approximately midway within each somite [94]. nt, neural tube. (B–D) Antibody staining of (B) acetylated tubulin, (C) Pcm1, and (D) merged image revealed that multiple basal bodies associate with each cilia tuft. (E) Antibody staining of acetylated tubulin (green) and Pcm1 (red) on transverse section of pronephric duct (counter-stained with WGA [blue]) revealed that the cilia tuft is within the lumen and there are multiple basal bodies within one cell. Arrows mark the cell membrane and arrowheads point to the individual Pcm1 staining in the neighboring cells. (F) Antibody staining of Pcm1 (green) and Zo-1 (red) on a transverse section of the pronephric duct revealed that multiple basal bodies are localized to the apical side of one cell. Arrows point to the Zo-1 staining. (G) Transmission electron microscope view of the lumen revealed that the cilia tuft contains at least 15 cilia in a closely organized manner and that individual primary cilia are present (arrows), all with the typical 9 + 2 structure (insert). Green lines demarcate cell borders. (H–K) Whole-mount (H, I, and K) and transverse section (J) in situ staining of sodium pump genes (H–J) *Na^+^, K^+^ ATPase α1a2* and (K) *Na^+^, K^+^ ATPase β1a* revealed that these genes are not expressed in some individual cells (demarcated by yellow dotted lines). (I) Magnified image of the box in (H). (L and M) Whole-mount (L) and transverse section (M) in situ staining of *rfx2* revealed that *rfx2* is expressed in individual cells. The red dotted line in (M) outlines the duct. (N) Whole-mount in situ staining of *centrin2* revealed that *centrin2* is expressed in the individual cells; the insert on the top is a magnified image of the box below. (O–Q) Fluorescent double in situ staining revealed that (O) *rfx2* and (P) *Na^+^, K^+^ ATPase β1a* are expressed in different cells. Arrows point to the cells that do not express *Na^+^, K^+^ ATPase β1a.* (Q) Merged image of (O) and (P); perceived costaining of *rfx2* and *Na^+^, K^+^ ATPase β1a* in some cells of the duct is an artifact caused by viewing at a single angle. Bar scale: 100 μm (A [bar in (D)]), 10 μm (B–D [bar in (D)], E, and F), 1 μm (G), 100 μm (H), 25 μm (I–K, [bar in (H)]), 70 μm (L), 25 μm (M, [bar in (L)]), 100 μm (N), 50 μm (O–Q [bar in (Q)]).

Acetylated tubulin staining revealed that stumpy single primary cilia are present in the pronephric duct as early as the 20 somite stage (ss) (unpublished data). Cilia tufts or multi-cilia appeared later and were fully formed by 36 h post-fertilization (hpf). These cilia tufts were located along the distal pronephric duct between the proximal pronephric duct and cloaca, corresponding to somites 8–14 (Figures 1A, S1, and S3) [11,27,28]. Pericentriolar material 1 (Pcm1) staining of 36-hpf embryos revealed that multiple basal bodies are associated with each cilia tuft (Figure 1B–1D) [29,30] and Pcm1 is colocalized with γ-tubulin at the apical site of epithelial cells (Figure S2). To determine if each cilia tuft is generated from a single cell, we used antibodies against a membrane marker, wheat germ agglutinin (WGA), and a tight junction marker, Zonula occludens-1 (Zo-1) [11,31]. Triple labeling of Pcm1, acetylated tubulin, and WGA demonstrated that cilia tufts are in the lumen of the duct and that multiple basal bodies are within one cell. Individual Pcm1 staining was also found in the neighboring cells corresponding to individual basal bodies of the primary cilia (Figure 1E). Immunostaining of Zo-1 and Pcm1 confirmed that multiple Pcm1-staining basal bodies are localized to the apical side of one cell in the pronephric duct (Figure 1F).

Cilia identity was further confirmed by transmission electron microscope imaging of a transverse section of the distal duct of 36-hpf embryos. We found two types of cells: cells with a cilia tuft of at least 15 cilia, and neighboring cells with a single primary cilium. All cilia tufts and primary cilia projected along the axis of the duct lumen and were of the typical 9 + 2 structure (Figure 1B and 1G), suggesting that they are motile [32]. Indeed, it was demonstrated that cilia in the zebrafish duct are motile, generating a corkscrew-like wave pattern in the duct lumen directed toward the cloaca [28].

In mammals, collecting ducts are composed of two major cell types: principal cells and intercalated cells [12]. Na^+^, K^+^ ATPases transport numerous solutes and water across epithelia [33] and are only expressed in the principal cells [34,35]. The zebrafish counterparts are expressed in the pronephric duct [36]. However, a meticulous examination of *Na^+^, K^+^ ATPase α1a2* and *Na^+^, K^+^ ATPase β1a* expression using in situ hybridization revealed that these genes are not expressed in all the duct cells (Figure 1H–1K). Some sodium pump-negative cells interpolated principal cells. To investigate their identity, we cloned the zebrafish homologs and examined the expression patterns of *pendrin1*, *pendrin2* [37,38], *rhcg* [39], and *vacuolar-type ATPase B* [40], all of which are marker genes in mammalian intercalated cells (see Materials and Methods). Although they were expressed in other tissues, none of these genes were expressed in the duct up to 72 hpf, suggesting that the sodium pump-negative cells are not intercalated cells (unpublished data). To determine whether they are multi-cilia cells, we cloned ciliogenic genes and analyzed their expression in the duct. Zebrafish *rfx2* is the homolog of *Caenorhabditis elegans daf-19,* which controls cilium formation in sensory neurons [41]. Zebrafish *centrin2* is the homolog of mouse *Centrin2,* which associates with centrosome-related structures of the basal bodies of the ciliated cells [42,43]. In addition to the ciliated tissues, including Kupffer's vesicle, olfactory pits, hair cells of the otic vesicle, and the neural tube (unpublished data) [44], *rfx2* and *centrin2* were expressed in a mosaic pattern in the duct at 36 hpf (Figure 1L–1N). Furthermore, fluorescent double in situ hybridization of *rfx2* and *Na^+^, K^+^ ATPase β1a* revealed a mutually exclusive pattern (Figure 1O–1Q). This indicates that multi-cilia cells and principal cells are two distinct cell populations in the zebrafish distal pronephric duct.

### *notch1a, notch3, jagged2a,* and *her9* are Expressed in the Duct

Notch signaling has been shown to be required for differentiation of ciliated cells in *Xenopus* skin [45] and in sensory patches of the zebrafish inner ear [46]. The mosaic pattern of multi-cilia cells and principal cells prompted us to explore whether Notch signaling is required for their differentiation in the pronephric duct.

**Figure 2:**
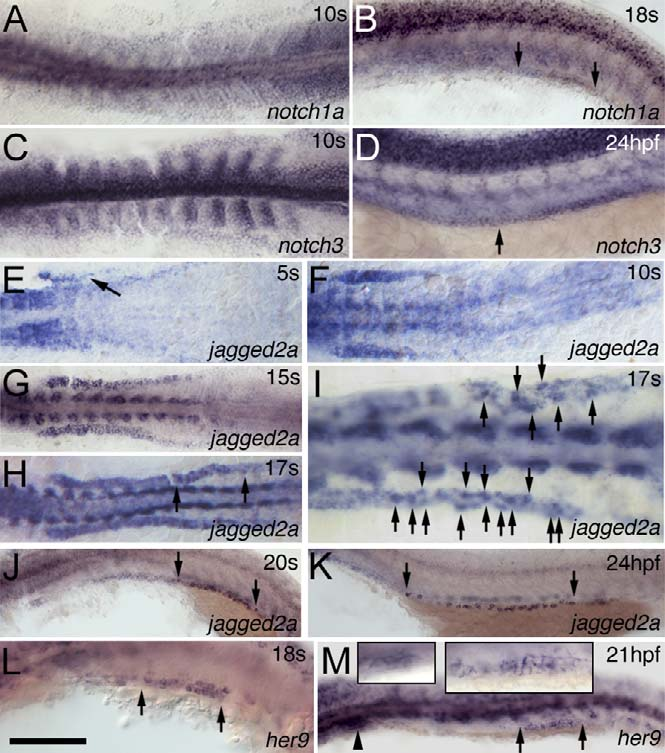
Dynamic Expression of Notch Components in the IM and Pronephric Duct (A–D) *notch1a* (A) and *notch3* (C) are expressed in the IM at 10 ss. *notch1a* (B) is expressed in the distal duct region from somite 10 to 14 (see also Figure S3B) at 18 ss, and *notch3* (D) is expressed in the whole duct from somite 3 to 20 at 24 hpf as indicated by the arrows. (E–G) *jagged2a* expression in the IM appears gradually from anterior to posterior from 5 ss (E) (as indicated by the arrow) to 10 ss (F), and reaches the posterior by 15 ss (G). (H–K) *jagged2a* expression is higher in some cells (arrows point to these cells in [I], which is magnified from [H]) than in neighboring cells in the distal duct at 17 ss (H and I), and transcription is limited to individual cells from 20 ss (J), to 24 hpf (K), to at least 36 hpf (unpublished data) in the demarcated region from somite 8 to 14 (see also Figure S3D and S3F) as indicated by arrows. (L and M) *her9* is expressed in the distal pronephric duct at 18 ss (L) from somite 10 to 12 (Figure S3H) and at 21 hpf (M). The arrowhead marks the glomerulus, and arrows demarcate the *her9* expression region. Left and right inserts in (M) are the magnified images in the glomerulus and distal duct, respectively. All embryos, anterior to the left. (A), (C), and (E–I) are dorsal views; the rest are lateral views. Bar scale: 200 μm (A, C, and E), 110 μm (B and D), 180 μm (F), 230 μm (G and H), 90 μm (I), 115 μm (J), 190 μm (K), and 100 μm (L and M).

Among four known Notch receptors, *notch1a* and *notch3* were found to be expressed in the intermediate mesoderm (IM) in early stages and later in the duct. *notch1a* was expressed in the IM from 1 ss and subsequently in the distal duct region at 18 ss (Figure 2A and 2B). *notch1a* expression, however, was not detected in the duct after 20 hpf. *notch3* was expressed in the IM from 1 ss onward and in the entire duct region, with a higher level of expression in the distal part, at 24 hpf (Figure 2C and 2D), where expression persisted until at least 48 hpf.

There are nine known zebrafish Notch ligands: *deltaA* [47], *deltaB* [48], *deltaC* [49], *deltaD* [50], *dll4* (M. M. and Y.-J. J., unpublished data), *jagged1a* (also known as *jagged1* or *serrateC*), *jagged1b* (also known as *jagged3* or *serrateA*), *jagged2a* (also known as *jagged2* or *serrateB*) [26,46,51], and *jagged2b* (M. M. and Y.-J. J., unpublished data). Of the ligands, *deltaC* is expressed in the anterior IM, presumably in the glomerulus, from 4 ss to 18 hpf [49], and *jagged1b* is expressed in the developing proximal tubule [51]. *jagged2a* expression in the IM appeared gradually from anterior to posterior, spanning from somite 3 to somite 13 at 15 ss (Figure 2E–2G). In the posterior IM, *jagged2a* expression displayed a salt-and-pepper-like pattern (a mixture of high- and low-expressing cells) from 17 ss to 20 ss (spanning from approximately somite 9 to somite 13) (Figure 2H and 2I). Beginning with 20 ss, *jagged2a* expression was limited to individual cells; this pattern persisted in the pronephric duct until at least 48 hpf (Figure 2J and 2K). *jagged2a* was also expressed in the proximal duct (Figure 2E–2J), and a description of its function there will be published elsewhere. Here, we only explore the function of *jagged2a* in the distal pronephric duct.

We further examined the expression of published Notch downstream targets, *hairy/enhancer of split related (her and hey)* genes, by doing in situ hybridization to detect *her1* [52], *her2* [53], *her3* [54], *her4* [55], *her6* [56], *her7* [57], *her8* [53], *her9* [58], *hey1, hey2,* and *heyL* [59]; or by checking the deposited expression patterns in the ZFIN database (http://www.zfin.org) of *her5, her12,* and *hes5* [60]. Only two of the *her* genes were expressed in the IM. *her6* appeared between the tailbud stage and 10 ss but expression was not maintained in later stages. *her9* expression was not detected in the duct domain before 15 ss (unpublished data). However, it was expressed in the distal duct from 17 ss. This correlates temporally with mosaic expression of *jagged2a* in the same region (Figures 2L and S3). Spotted and uneven *her9* expression persisted in the duct till about 21 hpf and disappeared afterwards (Figure 2M).

### Individual *jagged2a*-Positive Cells Are Multi-Cilia Cells

**Figure 3:**
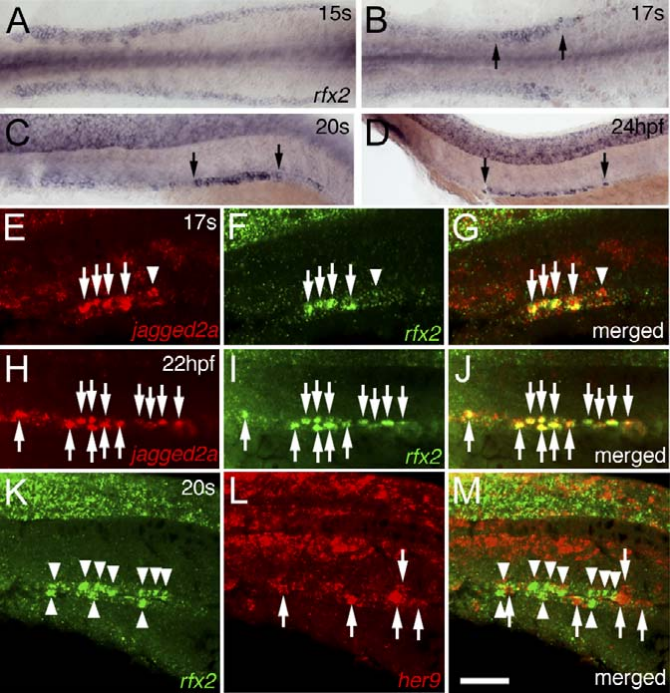
*jagged2a*-Expressing Cells in the Pronephric Duct are Multi-Cilia Cells (A–D) *rfx2* is expressed uniformly in the IM at (A) 15 ss, and expression is restricted to individual cells in the pronephric duct from (B) 17 ss onwards to (C) 20 ss and (D) 24 hpf. Arrows demarcate the distal duct region that contains *rfx2*-expressing cells. (E–J) Fluorescent double in situ hybridization of (E and H) *jagged2a* and (F and I) *rfx2* revealed that they are (G and J) colocalized in the distal pronephric duct of (E–G) 17-ss and (H–J) 22-hpf embryos. Arrows point to the cells that express *jagged2a* and *rfx2,* and arrowhead points to the cells that express *jagged2a* only. (K–M) Fluorescent double in situ hybridization of (K) *rfx2* and (L) *her9* revealed that they are expressed in the (M) alternate cells in the distal pronephric duct of 20-ss embryos. Arrowheads point to the *rfx2*-expressing cells and arrows point to the *her9*-expressing cells*.* All embryos, anterior to the left. (A) and (B) are dorsal views; (C–M) are lateral views. Bar scale: 135 μm (A), 150 μm (B), 100 μm (C), 120 μm (D), and 50 μm (E–M).

Since Jagged2a presumably starts signaling to the neighboring cells from 17 ss onwards, we investigated the onset of multi-cilia cell differentiation by examining the expression of *rfx2* and *centrin2* at earlier stages. Interestingly, *rfx2* expression in the IM and duct was similar to that of *jagged2a*. *rfx2* expression was uniform in all duct cells before 15 ss, which is consistent with the fact that all cells have cilia—either cilia tufts or a single cilium—in this kidney segment. Its expression was then limited to single cells in the distal duct from 17 ss until at least 36 hpf (Figure 3A–3D). Similarly, *centrin2* expression was limited to single cells from 20 ss onwards (unpublished data). These observations suggest that multi-cilia cells are *jagged2a*-expressing cells. Indeed, *jagged2a* and *rfx2* transcripts were colocalized in individual cells from 17 ss to at least 36 hpf (Figure 3E–3J). Furthermore, when we investigated whether *her9* is expressed in the same distal duct domain as *rfx2*, we found that although *her9* was expressed in the same domain, it was expressed primarily in non-*rfx2*-expressing cells (Figure 3K–3M).

### Jagged2a and Mib Regulate Cell Fate Differentiation through Lateral Inhibition

**Figure 4:**
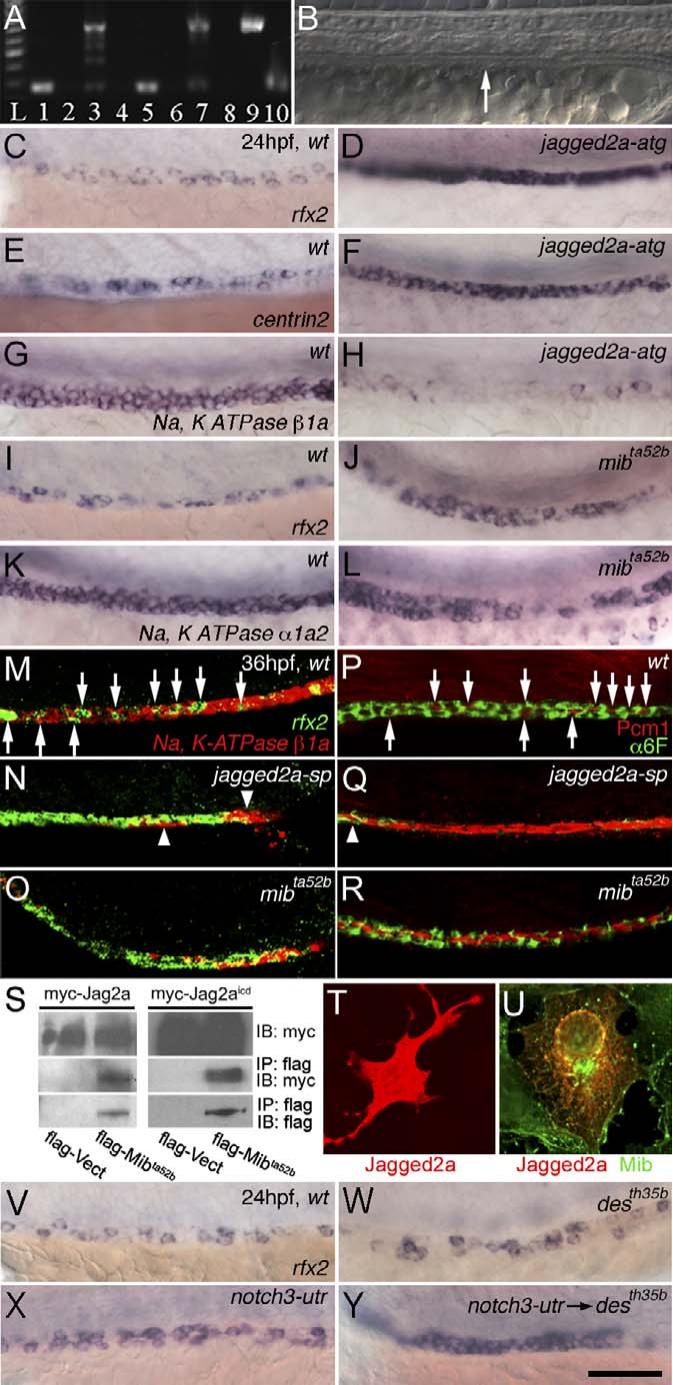
Multi-Cilia Cell Hyperplasia Is Due to Mib-Mediated Jagged2a Signaling Pathway via Notch1a and Notch3 Receptors (A) Effectiveness of splicing *jagged2a-sp* MO. RT-PCR of control embryos generates a 230-bp *jagged2a* fragment, bridging parts of exon 1 and exon 2 at 24 hpf (lane 1) and 48 hpf (lane 5). *jagged2a*-*sp* MO-injected embryos analyzed with the same primers at the same timepoints (lanes 3 and 7) show a larger amplicon of 708 bp caused by a nonsplicing intron 1, which encodes a premature stop codon. Lane 9 shows the amplicon from genomic DNA, and lane 10 shows the amplicon from *jagged2a* cDNA. No fragment can be amplified in the RT-PCR without reverse transcriptase in 24-hpf (lane 2) or 48-hpf (lane 6) wt embryos or in 24-hpf (lane 4) or 48-hpf (lane 8) *jagged2a-sp* MO-injected embryos. Lane L: 100-bp ladder. (B) Pronephric duct (arrow) integrity is not affected in *jagged2a* morphants. Panels C–L focus on the duct between somite 10 and 13. (C–H) Multi-cilia cell number is increased in (D and F) *jagged2a-atg* morphants compared to (C and E) wt embryos as shown by (C and D) *rfx2* and (E and F) *centrin2* expression at 24 hpf, but principal cell number is decreased in (H) *jagged2a-atg* morphants compared to (G) wt embryos as revealed by *Na^+^, K^+^ ATPase β1a* expression at 24 hpf. (I–L) Multi-cilia cell number is increased in (J) *mib^ta52b^* embryos compared to (I) wt embryos as shown by *rfx2* expression at 24 hpf, but principal cell number is decreased in (L) *mib^ta52b^* embryos compared to (K) wt embryos as revealed by *Na^+^, K^+^ ATPase α1a2* expression at 24 hpf. Panels M–R focus on the duct around somite 11 to 13. (M–O) Fluorescent double in situ hybridization of *rfx2* (green) and *Na^+^, K^+^ ATPase β1a* (red) in 36-hpf (M) wt embryos, (N) *jagged2a-sp* morphants, and (O) *mib^ta52b^* mutants shows multi-cilia cell hyperplasia in *jagged2a* morphants and *mib^ta52b^* mutants. Arrows point to the *rfx2*-expressing cells in the duct of (M) wt embryos; arrowheads point to the *Na^+^, K^+^ ATPase β1a*-expressing cells in the pronephric duct of (N) *jagged2a-sp* morphants. (P–R) Double immunohistochemistry of α6F (green) and Pcm1 (red) in 36-hpf (P) wt embryos, (Q) *jagged2a-sp* morphants, and (R) *mib^ta52b^* mutants shows multi-cilia cell hyperplasia in *jagged2a* morphants and *mib^ta52b^* mutants. Arrows point to the Pcm1 staining in the pronephric duct of (P) wt embryos; arrowheads point to α6F staining in the pronephric duct of (Q) *jagged2a-sp* morphants. (S) Immunoprecipitation of Myc-Jagged2a and Myc-Jagged2a^icd^ by Flag-Mib^ta52b^. IP, immunoprecipitation; IB, immunoblotting. (T–U) Expression of Myc-Jagged2a (T) and cotransfection of Myc-Jagged2a and Flag-Mib (U) in COS7 cells. (V–Y) Compared to (V) wt embryos, mild cilia cell hyperplasia is observed in (W) *notch1a (des^th35b^*) mutants and (X) *notch3-utr* morphants, while severe cilia cell hyperplasia is observed in (Y) *notch3-utr* MO-injected *notch1a (des^th35b^)* mutants as shown by *rfx2* expression at 24 hpf. All embryos, anterior to the left. Bar scale: 100 μm (B), 75 μm (C–L and V–Y), 50 μm (M–R), and 30 μm (T and U).

The above finding suggests that Jagged2a regulates differentiation of multi-cilia cells and principal cells. We designed morpholino antisense oligonucleotides (MOs) to knock down the function of Jagged2a, and used the *mib^ta52b^* mutant to study the function of the Jagged2a-Notch pathway in differentiation. In addition to two MOs, *jagged2a-atg* and *jagged2a-utr,* designed to be antisense to the *jagged2a* translation start site and the 5′ UTR, respectively, one MO *(jagged2a-sp)* was designed to block RNA splicing between exon 1 and intron 1. The *jagged2a-sp* MO effectively blocked splicing until at least 48 hpf (Figure 4A) and the *jagged2a-utr* MO was specific in a sequence-dependent manner (Figure S4A–S4F). *jagged2a* MOs did not affect duct development (Figure 4B). *jagged2a-atg* morphants displayed uniform *rfx2* (100%, *n* = 242) and *centrin2* (89%, *n* = 224) expression in almost all of the duct cells, in contrast to a mosaic pattern found in wild-type (wt) embryos (Figure 4C–4F). Similar results were found in *jagged2a-utr* morphants (Figure S4F; Table 1). Furthermore, we observed that Pcm1 and acetylated tubulin were significantly increased in *jagged2a-sp* morphants (100%, *n* = 7; Figure S5A–S5F). In contrast, *Na^+^, K^+^ ATPase β1a* expression was highly reduced in the duct of *jagged2a-atg* morphants at 24 hpf (100%, *n =* 22; Figure 4G and 4H) and 36 hpf (100%, *n* = 40; unpublished data). Similarly, we observed multi-cilia cell hyperplasia in *mib^ta52b^* mutants as evidenced by *rfx2* expression (Figure 4I and 4J) and immunostaining of acetylated tubulin and Pcm1 (Figure S5G–S5I). Statistically, *mib^ta52b^* mutants generated greater than 2-fold more multi-cilia cells than wt embryos (Table 1). Consistently, principal cells were decreased dramatically in *mib^ta52b^* mutants, as shown by *Na^+^, K^+^ ATPase α1a2* expression at 24 hpf (Figure 4K and 4L).

**Table 1:**
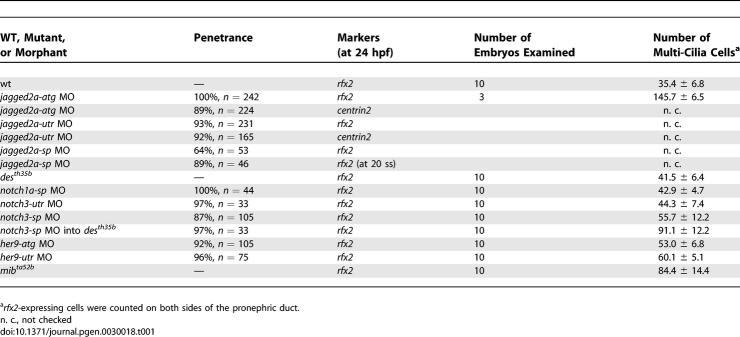
Statistical Analysis of Multi-Cilia Cell Number in WT Embryos, *des^th35b^* Mutants, *mib^ta52b^* Mutants, *jagged2a* Morphants, *notch1a* Morphants, and *notch3* Morphants

While the number of *rfx2*-expressing cells was dramatically increased in *jagged2a-sp* morphants and *mib^ta52b^* mutants (Table 1), only three to five *Na^+^, K^+^ ATPase β1a*-expressing cells were found in the *jagged2a-sp* morphants (89%, *n* = 19), and a dramatically decreased number of *Na^+^, K^+^ ATPase β1a*-expressing cells were found in *mib^ta52b^* mutants (Figure 4M–4O). Similarly, double immunostaining with α6F (raised against the chick α1 subunit of the Na^+^/K^+^ ATPase [61]) and Pcm1 showed that most of the duct cells adopted a multi-cilia cell fate and expressed Pcm1; only two to three cells were positive for the principal cell maker α6F in the distal duct of *jagged2a-sp* morphants (93%, *n =* 15), and principal cells were dramatically decreased in *mib^ta52b^* mutants (Figure 4P–4R). This observation suggests that the multi-cilia cell hyperplasia in *jagged2a* morphants and *mib^ta52b^* mutants is at the expense of the principal cells through lateral inhibition (see below). It is unlikely to be due to an inhibitory activity of Jagged2a on proliferation of multi-cilia cells, since there is no difference in cell proliferation between wt embryos and *jagged2a* morphants (unpublished data). The phenotypic severity of *mib^ta52b^* was not as strong as that of *jagged2a* morphants (Table 1), indicating that some residual Notch activity remains in *mib^ta52b^* mutants, as reported previously [20].

### Mib Binds and Internalizes Jagged2a in Cells

*mib^ta52b^* mutants display a global compromise in Notch activation, and *mib* was identified to encode an E3 ligase that activates Notch signaling by ubiquitylating and endocytosing Delta [20].

The phenotypic analysis of multi-cilia cells and principal cells in the duct suggests that *mib* genetically interacts with *jagged2a*. Since Delta has been shown to be a substrate of Mib and endocytosed after ubiquitylation [20], we asked if Mib physically interacts with Jagged2a, as shown for a human Mib paralog, Skeletrophin [21]. We checked the in vivo interaction of Jagged2a and Mib by immunoprecipitation and cotransfection experiments. Indeed, Mib bound to full-length Jagged2a and Jagged2a^icd^ (Figure 4S). Moreover, Myc-Jagged2 was localized to the cell surface (membrane) and cytoplasm when transfected alone (Figure 4T) and to the perinuclear granules when cotransfected with Flag-Mib (Figure 4U). The fact that Mib binds Jagged2a and facilitates its internalization suggests that Mib regulates Jagged2a in a way similar to Delta.

### Notch1a and Notch3 Receptors Function Redundantly in Jagged2a-Mediated Lateral Inhibition

In the duct, we observed slight multi-cilia cell hyperplasia in *notch1a*/*des (deadly seven)* mutants and *notch1a* morphants (Figure 4W; Table 1). *notch3-utr* MO and *notch3-sp* splicing MO against the exon 1-intron 1 boundary were designed; the former was specific in a sequence-dependent manner (Figure S4G–S4L) and the latter was able to induce splicing defects until at least 48 hpf (Figure S4M). More multi-cilia cells were found in *notch3* morphants (Figure 4X; Table 1). The stronger cilia phenotype seen in *notch3* morphants compared with that of *notch1a* mutants or morphants was consistent with the persistent *notch3* expression in the pronephric duct and also demonstrated that Notch3 plays a more important role than Notch1a.

Loss of function of a single Notch receptor resulted in a phenotype that was less severe than that of *jagged2a* morphants. This suggests that Notch1a and Notch3 act redundantly. In fact, 91.1 ± 12.2 multi-cilia cells were generated in *notch3-sp* MO-injected *des^th35b^* mutants in contrast to 42.9 ± 4.7 and 55.7 ± 12.2 multi-cilia cells in *notch1a-sp* and *notch3-sp* morphants, respectively (Figure 4V–4Y; Table 1). However, the multi-cilia cell phenotype in *notch3-sp* MO-injected *des^th35b^* mutants was not as severe as that of *jagged2a* morphants (Table 1). Consistently, *Na^+^, K^+^ ATPase β1a* down-regulation in *notch3-sp* MO-injected *des^th35b^* mutants was not as severe as that in *jagged2a* morphants (unpublished data). These data suggest that there may be a yet-unidentified Notch involved in this differentiation process.

### Her9 Acts Downstream in Jagged2a-Notch1a/Notch3-Mediated Lateral Inhibition

**Figure 5:**
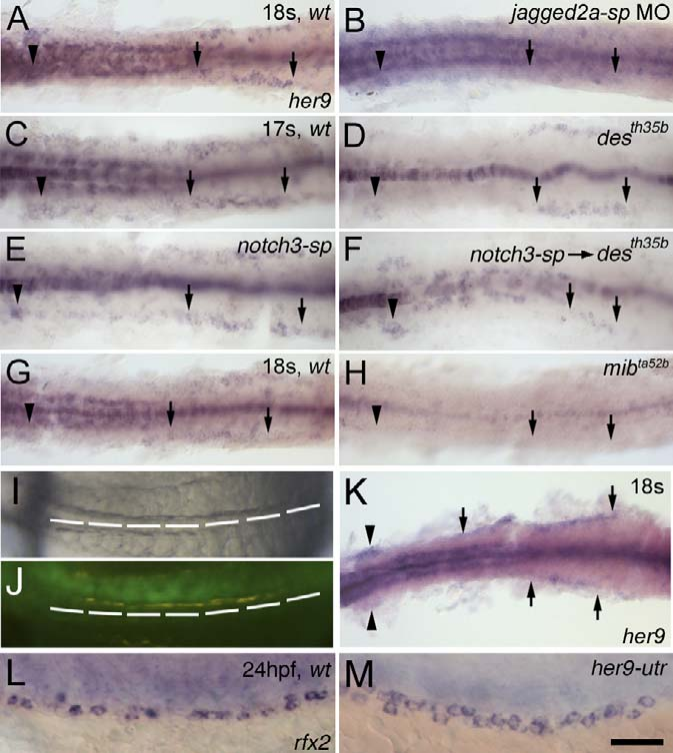
*her9* is a Downstream Target Gene of Jagged2a-Notch1a/Notch3 Signaling (A and B) Compared to (A) wt embryos, *her9* expression in the pronephric duct region at 18 ss is severely down-regulated in (B) *jagged2a-sp* morphants. (C–F) Compared to (C) wt embryos, *her9* expression in the pronephric duct region at 17 ss is mildly down-regulated in (D) *notch1a* (*des^th35b^*) mutants and (E) *notch3-sp* morphants, and is severely down-regulated in (F) *notch3-sp* MO-injected *notch1a* (*des^th35b^*) mutants. (G and H) Compared to (G) wt embryos, *her9* expression in the pronephric duct region at 18 ss is severely down-regulated in (H) *mib^ta52b^* mutants. (I and J) Coinjection of GFP mRNA (50 pg) and *notch1a^icd^* mRNA (100 pg) into one blastomere at the two-cell stage leads to (I) somite boundary disruption in the right half of the embryo, while somites on the left side are segmented properly. (J) GFP expression demonstrates that mRNA is localized to the right half of the embryo. (K) Compared to the left side of the embryo, *her9* expression in the duct (arrows) and glomerulus (arrowheads) is increased in the right side at 18 ss. (L and M) Compared to (L) wt embryos, the multi-cilia cell number is increased in (M) *her9-utr* morphants as shown by *rfx2* expression at 24 hpf. All embryos, anterior to the left. (A–K) are dorsal views; (L and M) are lateral views. Bar scale: 100 μm (A–J), 130 μm (K), and 50 μm (L and M).

The temporal and spatial expression of *her9* in the distal pronephric duct suggests that it is one of the downstream target genes of Jagged2a-Notch1a/Notch3 signaling. We asked whether activation of *her9* in the duct requires Jagged2a, Notch1a, and Notch3. *her9* expression in the pronephric duct was reduced in *jagged2a-sp* morphants (77%, *n* = 54; Figure 5A and 5B). While *her9* expression was slightly reduced in *des^th35b^*/*notch1a* mutants (Figure 5C and 5D) and in *notch3-sp* morphants (100%, *n* = 45; Figure 5E), its expression was almost completely lost in *notch3-sp* MO-injected *des^th35b^* mutants (94%, *n* = 36; Figure 5F). Similarly, its expression was almost completely lost in *mib^ta52b^* mutants (Figure 5G and 5H). We next examined whether *her9* is activated by Notch1a and Notch3. The constitutively active form, the intracellular domain (icd) of both Notch1a and Notch3, were used. *her9* expression was activated by both Notch1a^icd^ (22%, *n* = 98; Figure 5I–5K) and Notch3^icd^ (17%, *n* = 70; unpublished data). These experiments demonstrate that the activation of *her9* expression in the pronephric duct requires Notch1a and Notch3, in addition to Jagged2a and Mib.

We further studied *her9* function in the duct with *her9-atg* (effectiveness verified in [62]) and *her9-utr* MOs. *her9* morphants exhibited multi-cilia cell hyperplasia, as demonstrated by *rfx2* expression (Figure 5L and 5M; Table 1). The requirement of Jagged2a, Notch1a, and Notch3 for activation of *her9* expression in the duct, and the multi-cilia cell hyperplasia in *her9* morphants demonstrate that Her9 acts downstream of the Jagged2a-Notch1a/Notch3 pathway. However, multi-cilia cell hyperplasia in *her9* morphants was not as severe as that in *jagged2* morphants or *notch3-sp* MO-injected *des^th35b^* mutants (Table 1). One possibility is that Her9 is not completely knocked down by *her9* MOs, because of the potential negative autoregulatory feedback on the transcription by its protein, similar to Hes7 [63]. Alternatively, there may be more effector(s) working in parallel with Her9. The latter explanation is particularly likely, since the *her9* expression domain only partially overlaps with that of *jagged2a* (Figure S3).

### Multi-Cilia Cell Differentiation Requires Jagged2a from 17ss Onwards

**Figure 6:**
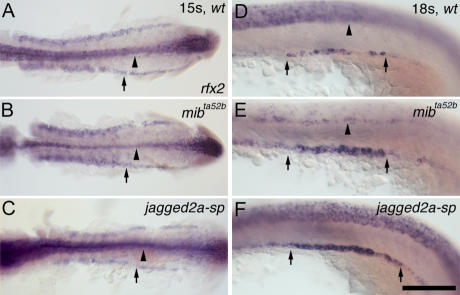
Multi-Cilia Cells Start to Differentiate as a Result of Jagged2a-Notch Signaling from 17 ss Onwards (A–C) *rfx2* expression in (A) wt embryos, (B) *mib^ta52b^* mutants, and (C) *jagged2a-sp* morphants at 15 ss. (D–F) *rfx2* expression in (D) wt embryos, (E) *mib^ta52b^* mutants, and (F) *jagged2a-sp* morphants at 18 ss. Arrows point to the *rfx2* expression in the (A–C) IM and (D–F) pronephric duct. *rfx2* staining in the neural tube (arrowheads) indicates the neurogenic phenotype in (B and E) *mib^ta52b^* mutants compared to that of (A and D) wt embryos. All embryos, anterior to the left. (A–C) are dorsal views; (D–F) are lateral views. Bar scale: 200 μm (A–C) and 100 μm (D–F).

*rfx2* and *jagged2a* displayed mosaic patterns from 17 ss onwards (Figure 3E and 3F); *her9* was expressed in the distal duct domain from 17 ss onwards (Figures 2L and 3L). Moreover, *her9*-expressing cells were not multi-cilia cells (Figure 3K–3M). The dynamic expression of these genes suggests that multi-cilia cells start to differentiate from 17 ss onwards. We next investigated whether Jagged2a-Notch signaling is required from as early as 17 ss. We found that *rfx2* expression is uniform in the IM in wt embryos (Figure 6A), *mib^ta52b^* mutants (Figure 6B), and *jagged2a-sp* morphants (91%, *n* = 33; Figure 6C) at 15 ss, while a neurogenic phenotype was obvious in *mib^ta52b^* mutants, indicating that multi-cilia cells do not start to differentiate before 15 ss. However, when *rfx2* expression was limited to individual cells at 18 ss in wt embryos (Figure 6D), *rfx2*-expressing cells were increased in the *mib^ta52b^* mutant (Figure 6E) and *jagged2a-sp* morphants (90%, *n* = 43; Figure 6F). Similarly, *her9* and *notch3* morphants exhibited multi-cilia cell hyperplasia from as early as 17 ss (unpublished data). These data indicate that Jagged2a-Mib-Notch3-Her9 is required for cell differentiation from as early as 17 ss.

### Duct Cells Adopt a Principal Cell Character at the Expense of Multi-Cilia Cells When Notch Is Constitutively Activated

**Figure 7:**
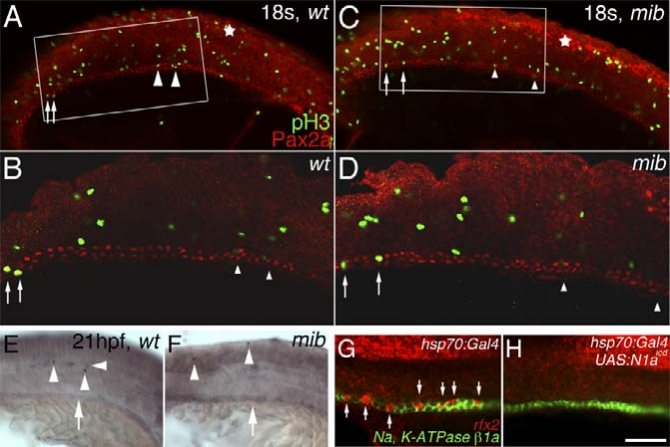
Notch-Dependent Binary Choice between Multi-Cilia cells and Principal Cells in the Pronephric Duct (A–D) double antibody staining of Pax2a (red) and phospho-histone-3 (pH3, green) of 18 ss (A and B) wt embryos and (C and D) *mib^ta52b^* mutants. In (A) and (C), some pH3-positive nuclei seem to overlap with Pax2a-positive nuclei in the pronephric duct (arrows and arrowheads). Higher magnification of the distal duct domain marked by the white box of the same (B) wt embryo and (D) *mib^ta52b^* mutant revealed that the pH3-positive nuclei indicated by arrowheads are not found in pronephric duct, while the nuclei indicated by arrows are overlapping with Pax2a-positive nuclei. A 3-D reconstruction of the domain revealed that the nuclei are not overlapping with Pax2a-positive nuclei (Videos S1 and S2). Pax2a staining in the neural tube (asterisk) indicates the neurogenic phenotype in (C) *mib^ta52b^* mutants compared to that of (A) wt embryos. Three wt embryos and four *mib^ta52b^* mutants were examined. In addition, three wt embryos and three *mib^ta52b^* mutants were sectioned, and all sectioned slices were examined. No proliferating cells were found in the duct domain (unpublished data). (E and F) Apoptosis assay with TUNEL method on (E) wt embryos and (F) *mib^ta52b^* mutants at 21 hpf. TUNEL staining was found in the somite and neural tube (arrowheads), while TUNEL staining was not found in the pronephric duct (arrows). The brown staining in the duct is background staining. Ten wt embryos and five *mib^ta52b^* mutants were examined. (G and H) Fluorescent double in situ hybridization of *rfx2* (red) and *Na^+^, K^+^ ATPase β1a* (green) in 24-hpf embryos demonstrated that multi-cilia cells interpolate principal cells in (G) heat-shocked *hsp70:Gal4* control embryos, while in (H) heat-shocked *hsp70:Gal4/UAS:myc*-*notch1a-intra* embryos, *Na^+^, K^+^ ATPase β1a* expression is robustly found in the duct cells but *rfx2* is not. Arrows point to *rfx2*-expressing cells. (A–D) are anterior to the right; (E–H) are anterior to the left. Bar scale: 100 μm (A and C), 50 μm (B and D), 100 μm (E and F), and 50 μm (G and H).

Multi-cilia cell hyperplasia is found in the mutants and morphants defective in the Jagged2a-Notch1a/Notch3-Her9 pathway. The increase of multi-cilia cells is most likely at the expense of principal cells, since no cell proliferation and apoptosis were detected in the duct of either wt or *mib^ta52b^* embryos (Figure 7A–7F; Videos S1 and S2). Thus, we demonstrated that Jagged2a-Notch1a/Notch3-Her9 is required for specification of multi-cilia cells and principal cells through a lateral inhibition mechanism. We next asked whether duct cells will adopt a principal cell fate if Notch is constitutively activated in the duct. We crossed transgenic lines *hsp70:Gal4* and *UAS:myc-notch1a-intra* and induced the expression of constitutively active Notch (Notch1a^icd^) by heat-shock from 6–8 ss. Fluorescent double in situ hybridization with *rfx2* and *Na^+^, K^+^ ATPase β1a* at 24 hpf revealed that multi-cilia cells (*rfx2*-expressing cells) interpolate principal cells (*Na^+^, K^+^ ATPase β1a*-expressing cells) in control embryos (heat-shocked *hsp70:Gal4* only) (100%, *n* = 4; Figure 7G), while principal cells are uniformly present at the expense of multi-cilia cells in *hsp70:Gal4*/*UAS:Notch1a^icd^* embryos (100%, *n* = 9; Figure 7H). This confirms that Notch signaling makes binary choices between multi-cilia cells and principal cells in the pronephric duct.

## Discussion

In this paper, we have shown that there are two major epithelial cell types found in the zebrafish distal pronephric duct. The mosaic pattern of multi-cilia cells and principal cells is controlled by Jagged2a/Notch-mediated lateral inhibition. Using available mutants and morphants deficient in genes functioning in Notch signaling, we demonstrated that one ligand, Jagged2a; two receptors, Notch1a and Notch3; and one downstream effector, Her9, are required for the differentiation and patterning of these two cell types. In addition, we showed that Mib is essential for this process, since it interacts with Jagged2a and facilitates Jagged2a internalization. In summary, our findings indicate a new function of Notch signaling in cell fate choice within a zebrafish kidney segment.

Interestingly, such a function of Jagged2-Notch signaling has not to our knowledge been found in mammals, although *Jagged2* is expressed in the postnatal murine kidney [64,65]. This may be due to the early lethality of *Jagged2* knockouts, which prevents the detection of such a function in metanephric kidneys. There are two zebrafish *Jagged2* homologs, *jagged2a* and *jagged2b*. Most likely, the subfunctionalization of these two genes makes it possible for us to identify the function of *jagged2a* in zebrafish pronephros. Our findings warrant further study of the role of *Jagged2* in mammalian kidneys by conditional knockouts.

The physiological functions of multi-cilia cells and principal cells are apparently different. While motile cilia on the apical side of the multi-cilia cells propel urea along the lumen of the pronephric duct [28], principal cells, which account for the majority of the cells in the kidney, reabsorb ions and other molecules according to fluid balance requirements. A plausible physiological significance of the interpolating pattern of multi-cilia cells and principal cells may be to coordinate the movement of the fluid and the process of reabsorption of the ions and other small molecules.

### Jagged2a-Mediated Lateral Inhibition of Multi-Cilia Cell Differentiation

**Figure 8:**
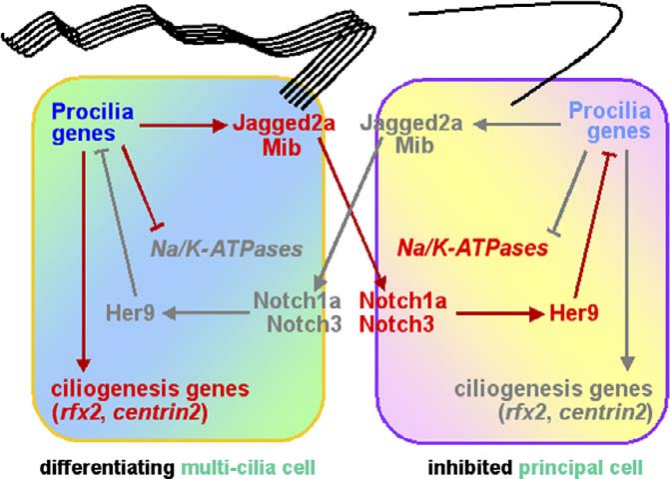
Model for the Role of Jagged2a/Notch Signaling during Differentiation of Multi-Cilia Cells and Principal Cells in the Zebrafish Distal Pronephric Duct Two adjacent cells of the developing pronephric duct are shown. The left cell, the winner in the lateral inhibition competition, differentiates as a multi-cilia cell (with cilia tuft and *rfx2* and *centrin2* expression, etc.), whereas the right cell is consequently inhibited and differentiates as a principal cell (with primary cilium and Na^+^, K^+^ ATPase expression). Activated components of the feedback regulatory system are highlighted in red, and inactive components are outlined in gray. Jagged2a is the sole ligand, Notch1a and Notch3 are two redundant receptors, and Her9 is one of the effectors that works downstream of the Notch receptors to prevent generating excessive multi-cilia cells at the expense of the principal cells. In this model, Mib affects Notch activity by interacting with Jagged2a and facilitating Jagged2a endocytosis in order to signal to neighboring cells. Procilia genes are hypothetical and have not been identified. In a manner similar to that of the proneural genes, procilia genes could encode bHLH transcription factors, stimulating expression of *jagged2a* and terminal differentiation (ciliogenesis) genes. Other components are mentioned in the text.

Notch signaling is used for binary cell fate specification in many developmental processes. Notch activation in the signal-receiving cells inhibits them from expressing a set of genes leading to one fate and diverts them to an alternative program of differentiation. Consistent with other recent expression studies in mice, chicks, and zebrafish [46,66,67], we found that the temporal and spatial expression patterns of *notch1a*, *notch3,* and *jagged2a* fulfill their predicted roles in a multi-cilia cell to principal cell inhibitory signaling process in the zebrafish distal pronephric duct (Figure 8). While the expression of Notch receptors is evident throughout the duct epithelium, *jagged2a* expression becomes restricted to developing multi-cilia cells (Figure 3G and 3J). In addition, *her9* is expressed unevenly within this domain and most *her9*-expressing cells are not colocalized with the multi-cilia cells (Figure 3M). Our observations support the notion that lateral inhibition regulates cell characters in the distal pronephric duct. In all of the mutants/morphants, Jagged2a-Notch signaling is thought to be blocked to different degrees, and many to almost all epithelial cells in the zebrafish pronephric duct become positive for *rfx2* and Pcm1, implying that they have adopted a multi-cilia cell—rather than a principal cell—character.

The studies shown here exemplify a very striking parallel between the role of Jagged2-Notch in the distal pronephric duct and the inner ear, and that of Delta-Notch signaling in neural tissue, the inner ear, and the intestine. In all of these cases, the obstruction of Notch signaling leads to a failure in lateral inhibition and to a great excess of one cell type at the expense of another. The supernumerary cell types are multi-cilia cells in the distal pronephric duct (this study), hair cells in the ear [46,67,68], neurons in the neural system ([48,69,70] and reviewed in [14]), and secretory cells in the gut [71]. Similar to Delta-Notch signaling, the blockage of Jagged2a-Notch signaling results in an up-regulation of *jagged2a* expression, implying that expression of *jagged2a* itself is negatively regulated by Notch activity. If a cell in the wt organism expresses *jagged2a,* thereby activating Notch in neighboring cells, it will not only inhibit these neighbors from adopting the primary fate, but it will also down-regulate their expression of *jagged2a*. This generates a feedback loop that, over time, tends to amplify differences between adjacent cells so as to create a mixture of different cell types (Figure 8; [72]).

### A Similar Mechanism for Other Similar Systems?

Multi-cilia cells are largely absent in mammalian kidneys, even though the primary cilium is present on principal cells of the tubule segment. We found interpolating multi-cilia cells and principal cells in the zebrafish distal pronephric duct. This mosaic cell pattern has been shown to be present in other anamniote vertebrates including marine teleosts [73], lampreys [74], and amphibians [75]. Notch signaling was required for the differentiation of speckled 4A6-positive cells in the posterior duct of *Xenopus* [22]. Our findings in zebrafish multi-cilia cells and the conserved pattern of cilia cells in amphibians [75] suggest that 4A6-positive cells are multi-cilia cells and that, in general, lateral inhibition may be involved in establishing the interpolating pattern of multi-cilia cells and principal cells in the ducts of anamniote vertebrates.

The renal collecting duct of mammalian kidneys comprises various kinds of intercalated cells (mediating acid and base transportation), principal cells (responsible for salt and water absorption), and inner medullary cells, which moderate all three types of transport. Inner medullary cells are “hybrid” cells—positive for both intercalated and principal cell markers [13,76,77]. Since *Jagged1* expression [23] and a similar mosaic pattern of intercalated cells and principal cells [13] were observed in the collecting ducts of mouse kidneys, it is tempting to speculate that Notch signaling is involved in the differentiation and patterning of these different cell types in the mammalian collecting duct.

The efferent duct transports material from the rete testis to the epididymis by motile cilia [78]. Similarly, multi-cilia cells and principal cells are found exhibiting a mosaic pattern in the efferent duct of reptile (turtle, [79]) and mammal (rat, [80]). It would be interesting to see how these two cell types differentiate and whether Notch signaling is involved in this differentiation process.

## Materials and Methods

### Zebrafish lines.

Fish were maintained and raised as described [81]. *mib^ta52b^, des^th35b^, hsp70:Gal4,* and *UAS:myc-notch1a-intra* mutants or transgenic lines were described before [69,82,83].

### Whole-mount and section in situ hybridization.

Primers based on zebrafish cDNAs or ESTs were designed (*rfx2,* forward: CTCACTCCTCACGCTCATCATC, reverse: CATAGGGTTTGAGCACCTGAT; *centrin2,* forward: TCAAAATGGCGTCCGGCTTC, reverse: GACACACTAGGTCTTAAAGG; *vhatpb1,* forward: TGCCTATGACAACAGAACG, reverse: CAAAGCACAGACGCTGTAAAC; *vhatpb1-2,* forward: ACTACCCTCTTTGTCTCGA, reverse: CGAAGCAAGTGGTCACATAC; *rhcg,* forward: GTAATCATGGAGACGGTCAG, reverse: GACAATGATCCGAACAGCAG; *pendrin1,* forward: CTCAACGAACGCTTCAAGAAG, reverse: CTGCTACATCCAGCAAGTAC; *pendrin2,* forward: CTGGATGTTGTGATGGAGC, reverse: AGAACACGCTCCAGTCTGAG; *slc4a2*/*ae2,* forward: GACTGCGCAACTTTGAGTCACGCAGTAGTG, reverse: CCAGGAATGAGGTCATACTGGCATTTGCATC; *ret1,* forward: GTTCACTACGTAACTTCCTG, reverse: CTATCGATTGTGTCCACG) and PCR-amplified products were purified and cloned into pGEMT-easy vector (Promega, http://www.promega.com). Together with *Na^+^, K^+^ ATPase α1a2* and *Na^+^, K^+^ ATPase β1a* [36], *her9* [58], *jagged2a* [46], *notch1a* [84], and *notch3* (previously annotated as *notch5* in [85] and changed according to the nomenclature in ZFIN, http://www.zfin.org), antisense probes were synthesized and whole-mount in situ hybridization was performed as described [86]. Whole-mount in situ embryos were embedded in Jung Tissue Freezing Medium (Leica, http://www.leica-microsystems.com), cryosectioned at 10 μm, and mounted in 70% glycerol. Images were taken with a Zeiss Axioplan microscope (http://www.zeiss.com) or a Leica MZ16 dissecting microscope equipped with SPOT INSIGHT (Diagnostic Instruments, http://www.diaginc.com).

### MO and mRNA injection.

To achieve maximal knockdown effect, 1.15 nl of serially diluted MOs (2.3 mM, 1.15 mM, 0.58 mM, and 0.29 mM; Gene Tools, http://www.gene-tools.com) was injected into embryos at the 1- to 2-cell stage. The maximal dosages that caused no obvious toxic effect on embryogenesis were as follows: *jagged2a-atg* (−22 to +3) MO: CATGCCGCCGATTTGATGTGTTATA, 2.30 pM; *jagged2a-utr* (−69 to −45) MO: ATGACCGGCGACAGGATCCTCCGTT, 0.29 pM; *jagged2a-sp* MO: AATCAGAGCTCTCACCTTCGTCCAC, 0.29 pM); *notch3-utr* (−76 to −51) MO: ACATCCTTTAAGAAATGAATCGGCG, 0.38 pM; *notch3-sp* MO: AAGGATCAGTCATCTTACCTTCGCT, 0.29 pM; *her9-atg* MO: CTCCATATTATCGGCTGGCATGATC, 1.15 pM [62]; *her9-utr* (−62 to −38) MO: AGTGAATATATTCCGTGTGTGGTTT, 0.29 pM; and *notch1a-sp* MO: GTAGTGTTAAACTGTTACCTTGTGC, 2.30 pM.

The knockdown efficacy of the splicing MOs was checked by reverse transcriptase PCR (RT-PCR) with the following primers: *jagged2a-sp* MO, forward: GGAATTGGCTCCCAATCGCGTGCCT, reverse: CCACCAAGAACGTCGGTAGATCCAG; *notch3-sp* MO, forward: CTGGAGGTATTTCGAGACGCACGGCAG, reverse: GCATCTTGAATCAATGCACATTCCTCC; and *notch1a-sp* MO, forward: CTTCTGCACTTTCTGGAGATTTAAAGAAG, reverse: GATGCTTCTCCGCTGGGCTTGTACTCGC and GCAACAAGTGACGCTCAAAGCGCAAGTTG (for spanning intron).

To examine the knockdown specificity of *jagged2a-utr* MO and *notch3-utr* MO, we cloned the 5′ UTR of both *jagged2a* (−130 to +3) and *notch3* (−226 to +3) to the EcoRI and XbaI sites of pCS2-XLT-GFP vector [87]. Plasmids were linearized with NotI, and mRNA syntheses were carried out with mMessage mMachine Kit (Ambion, http://www.ambion.com). Then, 250 pg of mRNA, 250 pg of mRNA with *utr-*MO, and 250 pg of mRNA with *mis-match-utr-*MO were injected into eggs at the one-cell stage. Green fluorescent protein (GFP) was examined under a Leica dissecting microscope, and images were taken by the equipped Nikon Digital (DXM1200F; Nikon, http://www.nikon.com) at 24 hpf. The sequences of the five-mismatch MOs (in lower case, designed by Gene Tools) are as follows:

*5mis-jagged2a-utr* (−69 to −45) MO: ATcACgGGCGAgAGGATCgTCCcTT and *5mis-notch3-utr* (−76 to −51) MO: AgATgCTTTAAcAAATcAATCGcCG.

We used mRNA of *notch1a^icd^* and *notch3^icd^* to examine the effect of Notch activation on *her9* expression*.* pCS2-*myc-notch1a^icd^* [55] and pCS2-*myc*-*notch3^icd^* [20] were linearized with NotI, and mRNA synthesis was carried out with mMessage mMachine Kit (Ambion). Then, 100 pg of *notch1a^icd^* or 100 pg of *notch3^icd^* mRNA was coinjected with 50 pg of GFP mRNA into one blastomere of two-cell-stage embryos. The mRNA-containing side was traced by following GFP expression, and mRNA functional expression was recognized by disrupted somite boundaries [88].

### Immunohistochemistry.

Whole-mount antibody staining was performed on embryos fixed in 4% PFA or methanol: DMSO (80:20) as described [11,89]. The following antibodies and their dilution were used: acetylated tubulin and γ-tubulin, 1:500 (Sigma-Aldrich, http://www.sigmaaldrich.com); anti-Pcm1, 1:200 [30]; monoclonal Zo-1, 1:20; monoclonal α6F, 1:5 (Developmental Studies Hybridoma Bank, http://www.uiowa.edu/~dshbwww); monoclonal phospohistone H3 (Ser10) (6G3), 1:40 (Cell Signaling Technology, http://www.cellsignal.com); rabbit anti-Pax2, 1:100 (Covance, http://www.covance.com); Alexa 488-goat anti-mouse and Alexa 568-goat anti-rabbit, 1:400 (Molecular Probes, http://probes.invitrogen.com); and Alexa 350-WGA, 1: 1,000 (Molecular Probes). The TUNEL assay was performed as described in the product manual of the In Situ Cell Death Detection Kit AP (Roche, http://www.roche-diagnostics.com). Whole-mount embryos were embedded in Jung Tissue Freezing Medium (Leica), cryosectioned at 10 μm, and mounted in FluorSave reagent (Calbiochem, http://www.emdbiosciences.com). Images were taken using a Zeiss Confocal LSM 510 or an Olympus Fluoview FV1000 microscope (http://www.olympusamerica.com), and 3-D movies were generated using FV10-ASW1.5 software.

### Transmission electronic microscopy.

Embryos were fixed with 2% paraformaldyhyde and 4% glutaraldehyde in 100 mM cacodylate buffer for 3 h and post-fixed with 2% osmium tetroxide in 100 mM sodium cacodylate buffer for 1 h at 4 °C. Embryos were then dehydrated through a series of 30%, 50%, 70%, 90%, and 100% ethanol, and finally in propylene oxide prior to infiltration with spurr resin [90]. Embryos were embedded in 100% spurr resin and polymerized at 65 °C overnight. Ultra-thin sections were cut on a Jung Reichert ultramicrotome (http://www.leica-microsystems.com) and examined with a transmission electronic microscope (JEM1010, JEOL, http://www.jeol.com) at 100 kV.

### Fluorescent double in situ hybridization.

The method was as previously described [91] except that substrates fluorescein-tyramide and Cy3-tyramide were respectively replaced with Alexa 488-tyramide and Alexa 568-tyramide (Molecular Probes). The substrates are diluted in amplification buffer/0.0015% H_2_O_2_ according to the product manual. Images were taken using a Zeiss Confocal LSM 510.

### Heat-shock treatment.

Embryos (6–8 ss) from *hsp70*:*Gal4* (homozygous) and *UAS:myc-notch1a-intra* (heterozygous) crossings were transferred to petri dishes with prewarmed (39 °C) egg water and incubated at 39 °C incubator for 40 min. Embryos were transferred to Petri dishes with 28 °C egg water afterwards and incubated at 28 °C until 24 hpf. The embryos in which Notch was activated were recognized by their short body axis [83].

### Plasmids, cell culture, transfection, immunoprecipitation, and Western blot analysis.

pCS2-myc-Jagged2a and pCS2-myc-Jagged2a^icd^ were cloned by in-frame fusion of *jagged2a* and *jagged2a^icd^* fragments to pCS2-myc vectors. The domain was predicted by the SMART program (http://smart.embl-heidelberg.de). pCS2-Flag-Mib was cloned by in-frame fusion of *mib* to the pCS2-Flag vector.

COS7 cells were transfected with 10 μg of plasmid DNA in 10-cm dishes using Dotap liposomal transfection reagent (Roche). Cells were harvested 2 d after transfection and lysed in lysis buffer (50 mM Tris-HCl [pH 8.0], 150 mM NaCl, 0.5% NP40, 0.5% deoxycholic acid, and 0.005% SDS). Lysates were centrifuged and the supernatant was incubated with Anti-Flag M2-Agarose Affinity Gel (Sigma-Aldrich) for 2 h. The beads were washed with lysis buffer seven times and with TBS (50 mM Tris-HCl [pH 8.0], and 150 mM NaCl) two times and boiled in SDS gel loading buffer. Eluted proteins were electrophoresed on an SDS-polyacrylamide gel and transferred to a nitrocellulose membrane (Stratagene, http://www.stratagene.com). Membranes were incubated with primary antibody (rabbit anti-Myc, 1: 1,000 [Santa Cruz Biotech, http://www.scbt.com]) for 2 h and secondary antibody (anti-rabbit-hrp, 1:5,000 [Dako, http://www.dako.com]) for 1 h. The signals were visualized with a chemiluminescence detection system (Pierce, http://www.piercenet.com). Then membranes were striped with 1× Re-Blot Plus Strong Solution (Chemicon, http://www.chemicon.com) and reblotted with rabbit anti-Flag, 1:1,000 (Sigma-Aldrich).

### Immunocytochemistry.

After a 24-h transfection, COS7 cells were fixed in methanol at 20 °C for 5 min and air-dried. Fixed cells were then incubated in blocking solution (10% goat serum in PBS) for 1 h, followed by staining with the appropriate primary antibodies (rabbit anti-Myc A14 [Santa Cruz Biotech] and mouse monoclonal anti-Flag M2 [Sigma-Aldrich], 1:1,000) in blocking solution for 1 h at room temperature. Subsequently, cells on coverslips were washed three times with PBS and incubated with Alexa 568-goat anti-rabbit antibody and Alexa 488-goat anti-mouse antibody (Molecular Probes). Coverslips were washed three times, mounted on glass slides, and observed under a Zeiss Confocal LSM 510.

## Supporting Information

Figure S1 Yolk Extension Spans from Somite 8, the Location of the Anterior Limit of Cilia Tufts Nomarski pictures of 48-hpf zebrafish embryos revealed that the yolk extension spans from somite 8 (arrowhead). The arrow points to the pronephric tubule ventral to somite 3 [92]. The first three somites are not in a regular chevron shape, in contrast to the posterior somites.
y, yolk; ye, yolk extension
(859 KB TIF)

Figure S2 Pcm1 and γ-Tubulin Colocalize Antibody staining of (A) Pcm1 and (B) γ-tubulin on transverse section of 36-hpf zebrafish pronephric duct revealed that they are (C) colocalized in the apical site of the duct epithelial cell. Arrowheads point to staining of the individual basal body, and arrows point to the staining of multiple basal body. Bar scale: 10 μm.
(461 KB TIF)

Figure S3 *notch1a*, *jagged2a,* and *her9* Are Expressed in the Distal Duct at the Time of Cell-Fate Determination (A and B) Fluorescent double in situ hybridization of *notch1a* and *myoD* [93] revealed that *notch1a* is expressed in the pronephric duct spanning from somite 10 to 14 (arrows) at 18 ss.
(C and D) Fluorescent double in situ hybridization of *jagged2a* and *myoD* revealed that mosaic *jagged2a* expression is found in the pronephric duct spanning from somite 8 to 14 (arrows) at 22 ss.
(E and F) Fluorescent double in situ hybridization of *jagged2a* and *myoD* revealed that *jagged2a*-expressing single cells are found in the pronephric duct spanning from somite 8 to 14 (arrows) at 24 hpf.
(G and H) Fluorescent double in situ hybridization of *her9* and *myoD* revealed that *her9* is expressed in the pronephric duct spanning from somite 10 to 12 (arrows) at 18 ss.
(I and J) Fluorescent double in situ hybridization of *jagged2a* (green), *slc4a2/ae2* (red, anterior), and *ret1* (red, posterior) revealed that *jagged2a*-expressing single cells are found in the distal duct between the proximal duct (marked by *slc4a2/ae2;* [27]) and the cloaca (marked by *ret1;* [11]). Small arrows demarcate the *jagged2a*-expressing single cell domain, arrowheads demarcate the *slc4a2*/*ae2*-expressing domain, and big arrows demarcate the *ret1*-expressing domain.
(3.6 MB TIF)

Figure S4 Specificity of *jagged2a-utr* and *notch3-utr* Morpholinos on Targeting the 5′ UTR of *jagged2a* and *notch3* and the Effectiveness of the *notch3-sp* Splicing Morpholino (A–C) Specificity of the *jagged2a* morpholino. (A) Injection of *jagged2a-utr-*GFP mRNA at 250 pg produced green fluorescence, (B) coinjection of 0.29 pM *jagged2a-utr-*MO with 250 pg of *jagged2a-utr-*GFP mRNA inhibited GFP production, and (C) coinjection of 0.29 pM *5mis-match*-*jagged2a-utr-*MO with 250 pg of *jagged2a-utr-*GFP mRNA did not inhibit its production.
(D–F) Multi-cilia cell probed with *rfx2* at 24 hpf in (D) wt embryos, (E) *jagged2a-utr* morphants, and (F) *5mis-match-jagged2a-utr* morphants. Note that the number of multi-cilia cells was increased in *jagged2a-utr* morphants (Table 1, 93%, *n* = 231) but not in *5mis-match-jagged2a-utr* morphants (97%, *n* = 35).
(G–I) Specificity of the *notch3* morpholino. (G) Injection of *notch3-utr-*GFP mRNA at 250 pg produced green fluorescence, (H) coinjection of 0.38 pM *notch3-utr-*MO with 250 pg of *notch3-utr*-GFP inhibited GFP production, and (I) coinjection of 0.38 pM *5mis-match-notch3-utr-*MO with 250 pg of *notch3-utr*-GFP did not inhibit its production.
(J–L) Multi-cilia cells probed with *rfx2* at 24 hpf in (J) wt embryos, (K) *notch3-utr* morphants, and (L) *5mis-match-notch3-utr* morphants. Note that the number of multi-cilia cells was increased in *notch3-utr* morphants (Table 1, 97%, *n* = 33) but not in *5mis-match-notch3-utr* morphants (100%, *n* = 30).
(M) Molecular analysis of the effectiveness of the *notch3-sp* splicing morpholino. RT-PCR of ten embryos generates a 320-bp *notch3* fragment in control embryos, bridging part of exon 1 to part of exon 2 at 24 hpf (lane 3) and 48 hpf (lane 4). *notch3-sp* morpholino-injected embryos analyzed with the same primer sets at 24 hpf (lane 1) and 48 hpf (lane 2) show a larger amplicon of 1,800 bp caused by a nonsplicing of intron 1 and other aberrant splicing variants. Lane L: 100-bp ladder.
Bar scale: 1,000 μm (A–C and G–I) and 100 μm (D–F and J–L).
(2.3 MB TIF)

Figure S5 Pronephric Duct Phenotype in *jagged2a* Morphants and *mib^ta52b^* Mutants Antibody staining of (A, D, and G) acetylated tubulin and (B, E, and H) Pcm1 shows that multi-cilia cell number is increased in (D–F) *jagged2a-sp* morphants and (G–I) *mib^ta52b^* mutants compared to (A–C) wt embryos at 36 hpf. Bar scale: 50 μm.
(498 MB TIF)

Video S1 Reconstruction (3-D) of pH3 and Pax2a Antibody Staining in the Distal Pronephric Duct of WT Embryos Reconstruction (3-D) of pH3 (green) and Pax2a (red) antibody staining in the distal pronephric duct of the embryo shown in Figure 7A reveals that pH3 nuclei are not localized to the pronephric duct domain of wt embryos at 18 ss. Note that the putative colocalized nuclei (yellow) turn partially to completely green at some rotating angles, suggesting that pH3-positive and Pax2a-positive cells are not colocalized, but in close vicinity to one another. Embryo is in lateral view, rotating around the dorsoventral axis.
(747 KB AVI)

Video S2 Reconstruction (3-D) of pH3 and Pax2a Antibody Staining in the Distal Pronephric Duct of *mib^ta52b^* Mutants Reconstruction (3-D) of pH3 (green) and Pax2a (red) antibody staining in the distal pronephric duct of the embryo shown in Figure 7C reveals that pH3 nuclei are not localized to the pronephric duct domain of *mib^ta52b^* mutants at 18 ss. Note that the putative colocalized nuclei (yellow) turn partially to completely green at some rotating angles, suggesting that pH3-positive and Pax2a-positive cells are not colocalized, but in close vicinity to one another. Embryo is in lateral view, rotating around the dorsoventral axis.
(1.1 MB AVI)

## Accession Numbers

The National Center for Biotechnology Information (http://www.ncbi.nlm.nih.gov) accession numbers for the genes discussed in this paper are *centrin2,*CF269323; *jagged1a* (also known as *jagged1* or *serrateC*), AY221107; *jagged1b* (also known as *jagged3* or *serrateA*), AY221106; *jagged2a* (also known as *jagged2* or *serrateB*), AF090432; *pendrin1,* BC054604; *pendrin2,* BC054629; *ret1,* NM_181662; *rfx2,* BC090314; *rhcg,* AF398238; *slc4a2*/*ae2,* AY876015; *vhatpb1,* AF472614; and *vhatpb1-2,* AF472615.

## Abbreviations

GFP: green fluorescent protein
hpf: hours post-fertilization
[protein]^icd^: [protein] intracellular domain
IM: intermediate mesoderm
MO: morpholino antisense oligonucleotide
RT-PCR: reverse transcriptase PCR
ss: somite stage
wt: wild-type
