# Supplementary figures and images for: Jagged2a-Notch Signaling Mediates Cell Fate Choice in the Zebrafish Pronephric Duct
Source: PLoS Genet. 2007 Jan 26;3(1):e18. doi: 10.1371/journal.pgen.0030018 (PMC1781496; doi:10.1371/journal.pgen.0030018)

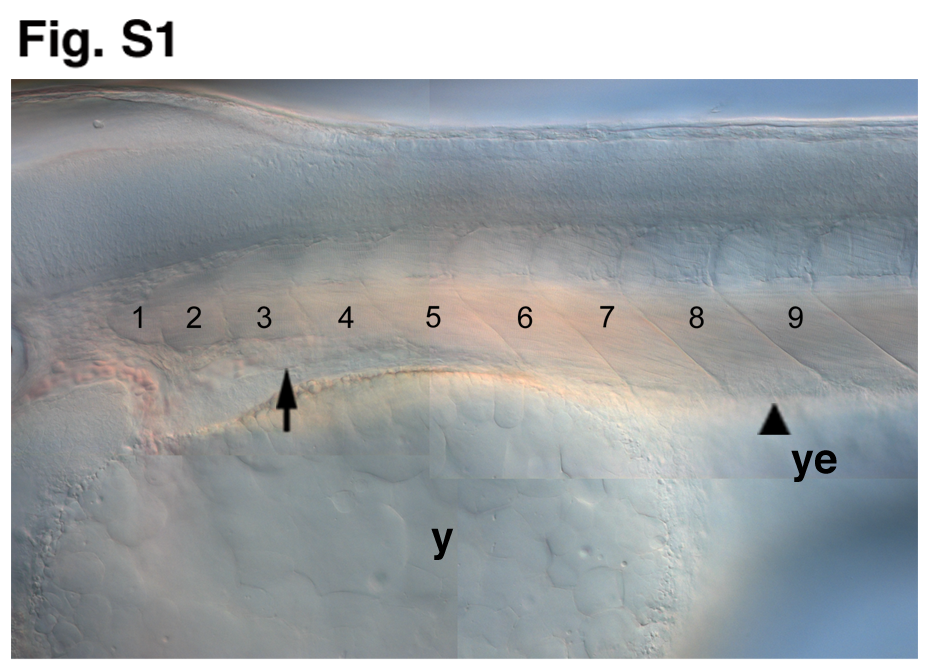

Supplement: Figure S1 — Nomarski pictures of 48-hpf zebrafish embryos revealed that the yolk extension spans from somite 8 (arrowhead). The arrow points to the pronephric tubule ventral to somite 3 [92]. The first three somites are not in a regular chevron shape, in contrast to the posterior somites. y, yolk; ye, yolk extension (859 KB TIF) [file pgen.0030018.sg001.tif]

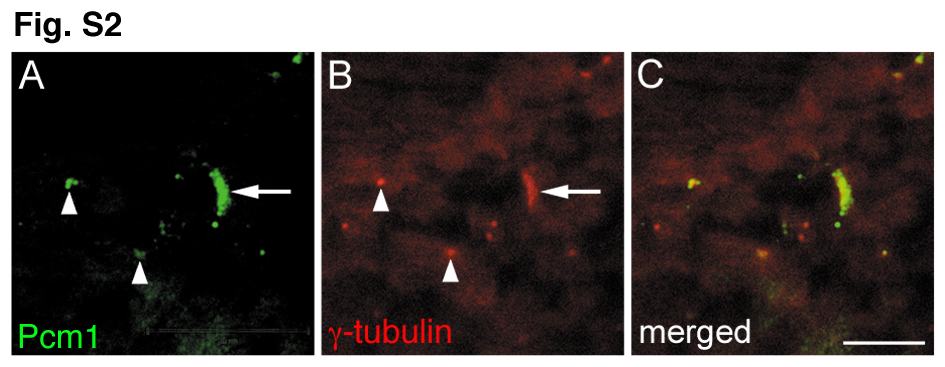

Supplement: Figure S2 — Antibody staining of (A) Pcm1 and (B) γ-tubulin on transverse section of 36-hpf zebrafish pronephric duct revealed that they are (C) colocalized in the apical site of the duct epithelial cell. Arrowheads point to staining of the individual basal body, and arrows point to the staining of multiple basal body. Bar scale: 10 μm. (461 KB TIF) [file pgen.0030018.sg002.tif]

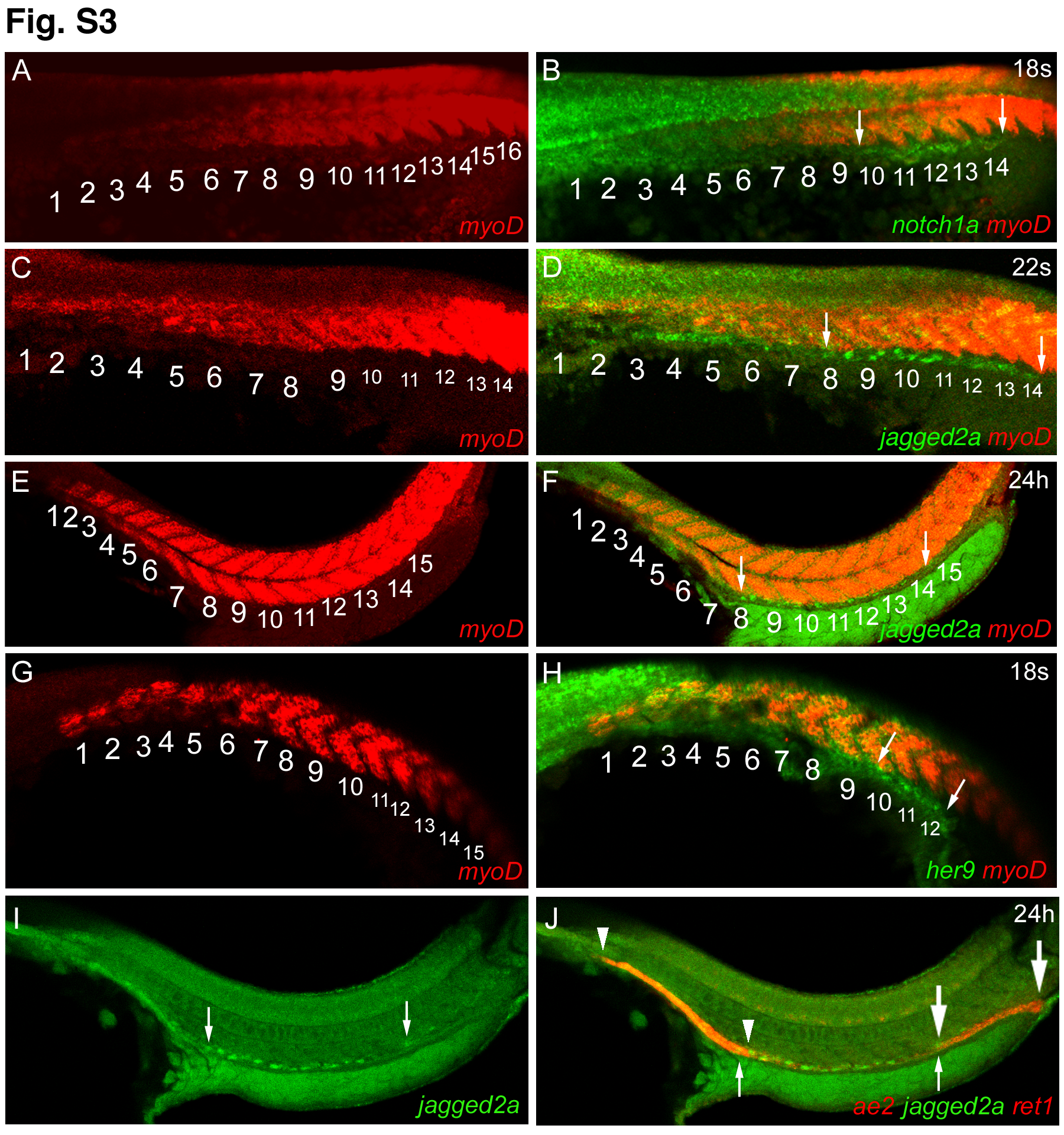

Supplement: Figure S3 — (A and B) Fluorescent double in situ hybridization of notch1a and myoD [93] revealed that notch1a is expressed in the pronephric duct spanning from somite 10 to 14 (arrows) at 18 ss. (C and D) Fluorescent double in situ hybridization of jagged2a and myoD revealed that mosaic jagged2a expression is found in the pronephric duct spanning from somite 8 to 14 (arrows) at 22 ss. (E and F) Fluorescent double in situ hybridization of jagged2a and myoD revealed that jagged2a-expressing single cells are found in the pronephric duct spanning from somite 8 to 14 (arrows) at 24 hpf. (G and H) Fluorescent double in situ hybridization of her9 and myoD revealed that her9 is expressed in the pronephric duct spanning from somite 10 to 12 (arrows) at 18 ss. (I and J) Fluorescent double in situ hybridization of jagged2a (green), slc4a2/ae2 (red, anterior), and ret1 (red, posterior) revealed that jagged2a-expressing single cells are found in the distal duct between the proximal duct (marked by slc4a2/ae2; [27]) and the cloaca (marked by ret1; [11]). Small arrows demarcate the jagged2a-expressing single cell domain, arrowheads demarcate the slc4a2/ae2-expressing domain, and big arrows demarcate the ret1-expressing domain. (3.6 MB TIF) [file pgen.0030018.sg003.tif]

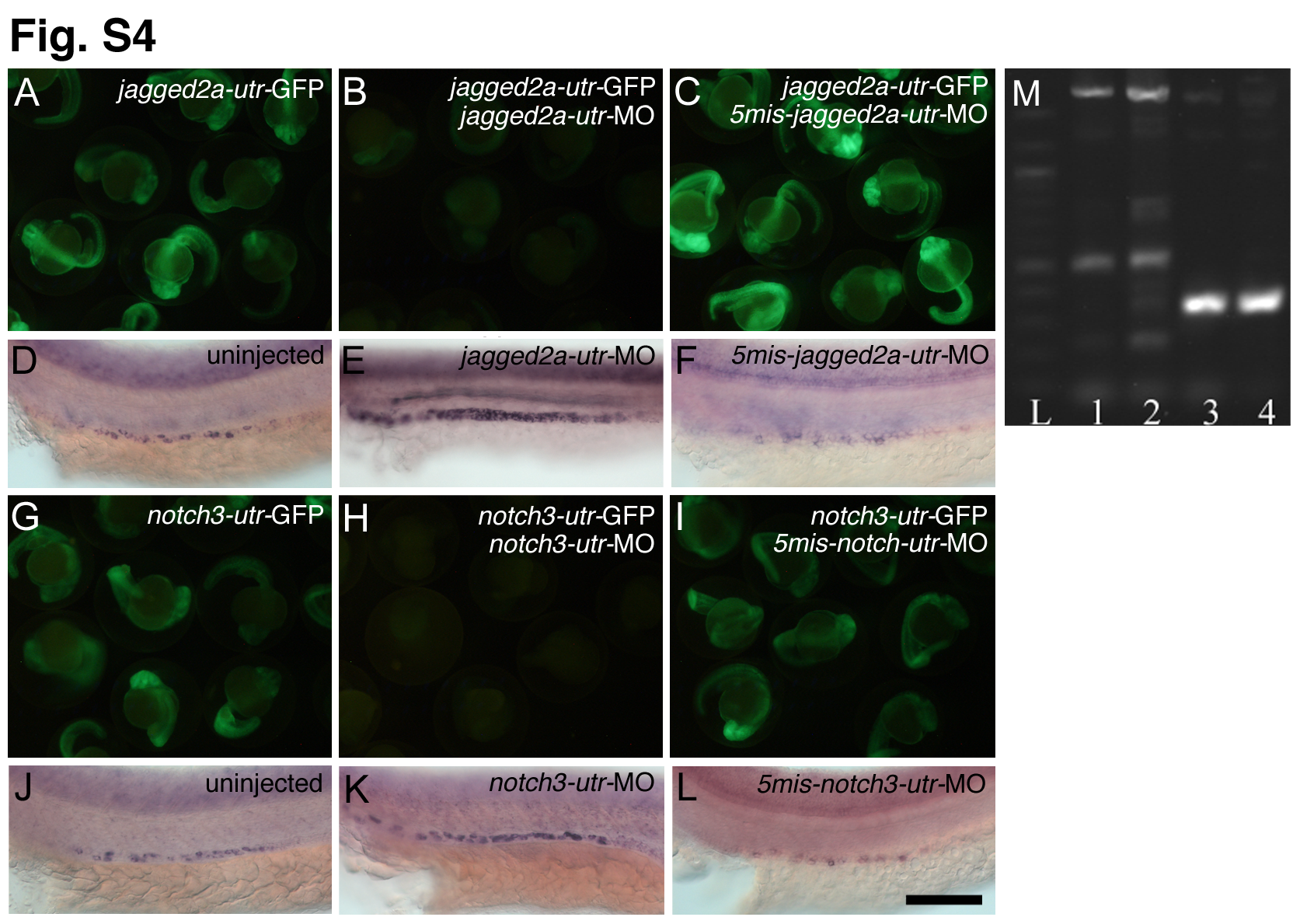

Supplement: Figure S4 — (A–C) Specificity of the jagged2a morpholino. (A) Injection of jagged2a-utr-GFP mRNA at 250 pg produced green fluorescence, (B) coinjection of 0.29 pM jagged2a-utr-MO with 250 pg of jagged2a-utr-GFP mRNA inhibited GFP production, and (C) coinjection of 0.29 pM 5mis-match-jagged2a-utr-MO with 250 pg of jagged2a-utr-GFP mRNA did not inhibit its production. (D–F) Multi-cilia cell probed with rfx2 at 24 hpf in (D) wt embryos, (E) jagged2a-utr morphants, and (F) 5mis-match-jagged2a-utr morphants. Note that the number of multi-cilia cells was increased in jagged2a-utr morphants (Table 1, 93%, n = 231) but not in 5mis-match-jagged2a-utr morphants (97%, n = 35). (G–I) Specificity of the notch3 morpholino. (G) Injection of notch3-utr-GFP mRNA at 250 pg produced green fluorescence, (H) coinjection of 0.38 pM notch3-utr-MO with 250 pg of notch3-utr-GFP inhibited GFP production, and (I) coinjection of 0.38 pM 5mis-match-notch3-utr-MO with 250 pg of notch3-utr-GFP did not inhibit its production. (J–L) Multi-cilia cells probed with rfx2 at 24 hpf in (J) wt embryos, (K) notch3-utr morphants, and (L) 5mis-match-notch3-utr morphants. Note that the number of multi-cilia cells was increased in notch3-utr morphants (Table 1, 97%, n = 33) but not in 5mis-match-notch3-utr morphants (100%, n = 30). (M) Molecular analysis of the effectiveness of the notch3-sp splicing morpholino. RT-PCR of ten embryos generates a 320-bp notch3 fragment in control embryos, bridging part of exon 1 to part of exon 2 at 24 hpf (lane 3) and 48 hpf (lane 4). notch3-sp morpholino-injected embryos analyzed with the same primer sets at 24 hpf (lane 1) and 48 hpf (lane 2) show a larger amplicon of 1,800 bp caused by a nonsplicing of intron 1 and other aberrant splicing variants. Lane L: 100-bp ladder. Bar scale: 1,000 μm (A–C and G–I) and 100 μm (D–F and J–L). (2.3 MB TIF) [file pgen.0030018.sg004.tif]

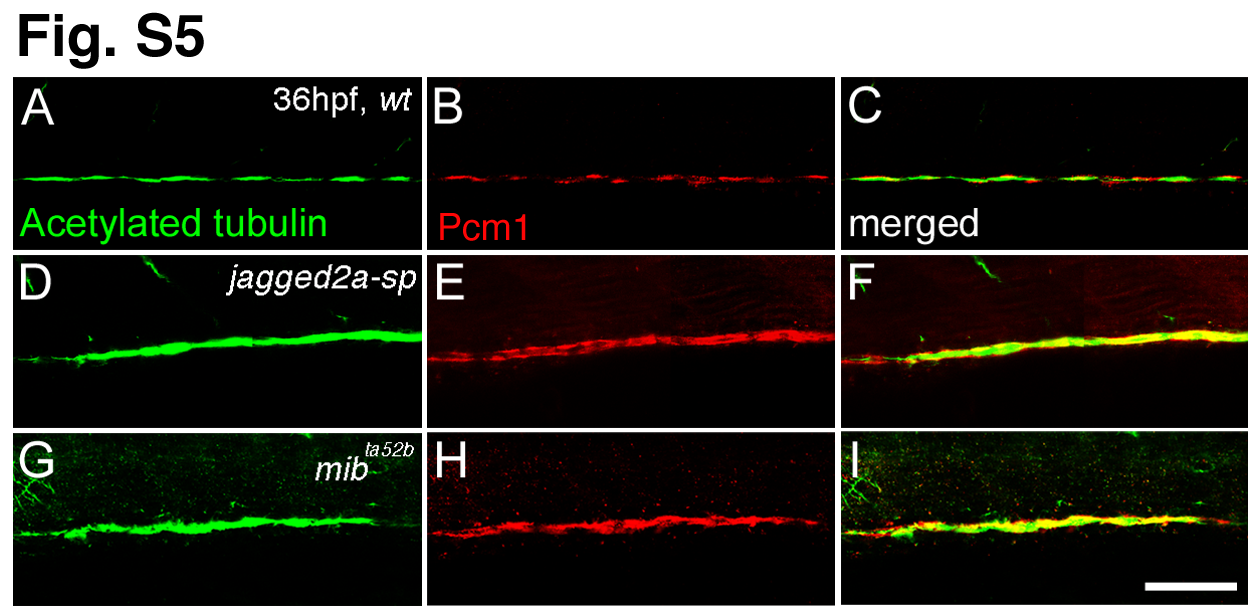

Supplement: Figure S5 — Antibody staining of (A, D, and G) acetylated tubulin and (B, E, and H) Pcm1 shows that multi-cilia cell number is increased in (D–F) jagged2a-sp morphants and (G–I) mibta52b mutants compared to (A–C) wt embryos at 36 hpf. Bar scale: 50 μm. (498 MB TIF) [file pgen.0030018.sg005.tif]
